# Quantification by real-time PCR of *Trypanosoma cruzi* DNA in samples of *Triatoma infestans* used in xenodiagnosis of chronic Chagas disease patients

**DOI:** 10.1186/s13071-016-1664-5

**Published:** 2016-07-04

**Authors:** Miguel Saavedra, Inés Zulantay, Werner Apt, Juan Castillo, Eduardo Araya, Gabriela Martínez, Jorge Rodríguez

**Affiliations:** Laboratorio de Parasitología Básico-Clínico, Programa de Biología Celular y Molecular, Instituto de Ciencias Biomédicas, Facultad de Medicina, Universidad de Chile, Santiago, Chile; Escuela de Salud Pública, Facultad de Medicina, Universidad de Chile, Santiago, Chile

**Keywords:** *Trypanosoma cruzi*, *Triatoma infestans*, Xenodiagnosis, Real-time PCR, Chronic Chagas disease, Parasite burden

## Abstract

**Background:**

*Trypanosoma cruzi* multiplies and differentiates in the digestive tract of triatomine insects. Xenodiagnosis (XD) is a parasitological tool in which the insect vectors acts as a biological culture medium to amplify and detect *T. cruzi* infection in mammals. The sensitivity of XD has been overcome by the application of PCR in fecal samples (FS) of XD (PCR-XD). In this study, *T. cruzi* amplified in *Triatoma infestans* fed by XD on individuals with chronic Chagas disease (CChD) is quantified by real-time PCR (qPCR-XD).

**Findings:**

Under informed consent, 100 individuals were evaluated. In 21 of them XD, PCR-XD and qPCR-XD were positive. For the contrary, 79 were negative XD. In 58 (73.4 %) and 66 cases (83.5 %) of them, PCR-XD (Fisher’s exact test *P* = 0.005) and qPCR-XD (Fisher’s exact test: *P* = 0.037) respectively, were positive. In cases with positive XD, qPCR-XD allowed to establish that in 9/21 cases (42.9 %) the parasite burden fluctuated between 100 and 1,000 par. eq./ml. Otherwise, in 32/79 (40.5 %) cases with negative XD, a parasite burden between 1 and 10 par. eq./ml was determined. All samples showed amplification of exogenous internal control (X12, Ct average: 31.8), so problems in the DNA extraction (excess or loss of genetic material), unspecific amplification and/or inhibition in qPCR-XD reactions were ruled out. Additionally, in all the patients qPCR in blood (qPCR-B) was performed. In the cases with positive XD, the concordance between the positivity of qPCR-XD and qPCR-B was 100 %, nevertheless, the parasite burden in blood was lower and different than XD (Chi-square test: *χ*^*2*^ = 91.82*, df* = 5, *P* = 0.0001). In the cases with negative XD the ranges of qPCR-XD and qPCR-B were similar (Chi-square test: *χ*^*2*^ = 6.71, *df* = 5, *P* = 0.1520).

**Conclusions:**

This study allowed the detection and quantification of *T. cruzi* by qPCR-XD in FS of *Tr. infestans* fed on patients with CChD. The highest parasite burden was observed in positive XD cases. qPCR-XD could be used in different studies related with the complex *T. cruzi*-vector-host interactions.

**Electronic supplementary material:**

The online version of this article (doi:10.1186/s13071-016-1664-5) contains supplementary material, which is available to authorized users.

## Background

Chagas disease (ChD) is a zoonosis caused by the parasite *Trypanosoma cruzi* endemic to the Americas, where it is vector-borne by triatomine bugs (subfamily Triatominae). An estimated 5.7 million people are infected with ChD in 21 Latin American countries [42]. In 1999, Chile was declared free of transmission of *T. cruzi* by *Triatoma infestans*; however, it is estimated that 140,000 people are infected with *T. cruzi* in the country, mostly without tripanocidal treatment [36, 37, 50]. Human ChD has two clinical phases. The acute phase appears just after infection while the chronic phase may last several years. After a long asymptomatic phase, around 30 % of infected individuals develop chronic disease with severe damage to the heart and digestive system [45]. In control treatment of an individual with ChD (pre and post-therapy), it is essential to determine the parasitological condition [38]. The classical xenodiagnosis (XD) [8, 32] has been used as a tool of selection for specific treatment and its posterior evaluation [2, 7, 16], searching for the most effective triatomine species [9], isolation and characterization of *T. cruzi* DTUs [13, 26, 33, 34, 47], comparison of its sensitivity with other procedures for the detection of *T. cruzi* in bloodstream [6, 51] and relation parasite cycle-vector [18, 41]. In most of these studies, the result of XD has been expressed in qualitative terms, i.e. fundamentally based on the microscopic observation of mobile tripomastigote forms of *T. cruzi* [11, 23, 39]. The high copy numbers of repetitive sequences contained in nuclear and kinetoplast DNA of *T. cruzi* [16, 17] has allowed the successful application of PCR to detect the parasite. The XD has less sensitivity than PCR but the combination with PCR (qualitative PCR-XD) in different biological samples and hosts, improves its sensitivity [15, 19–22, 28, 29, 35, 47, 51]. The objective of this study is to detect and quantify *T. cruzi* in *Tr. infestans* fed by XD on individuals with CChD by real-time PCR (qPCR-XD).

## Methods

### Study population

A total of 100 individuals with CChD, 31 men and 69 women (average age 49.6 years; range 10–79) were identified during a serological survey performed between 2012 and 2014. All participants were from rural and urban localities of the Provinces of Limarí and Choapa IV Region, Coquimbo, located between 29°02’S and 32°16’S in an area of transverse valleys of Chile. With a population of 718,717 inhabitants, 21.9 % come from rural settings [30]. *T. cruzi* infection was determined previously by Chagatek ELISA (Biomerieux Corp., Buenos Aires, Argentina) and IFI-IgG (in house developed). Previous clinical evaluations found that all of the cases were asymptomatic at baseline and had not received tripanocidal drugs.

### Xenodiagnosis

Xenodiagnosis was carried out using two cages, each containing seven uninfected III or IV instar nymphs of *Tr. infestans*. The method, criteria of positivity and the preparation of fresh fecal samples (FS) for PCR-XD are described in Saavedra et al. [46]. Before FS-DNA isolation an exogenous internal control (EIC) was added [4]. DNA purification was performed as described [2].

### PCR-xenodiagnosis (PCR-XD)

PCR-XD was performed in triplicate using oligonucleotides 121 and 122, which anneal to the four conserved regions present in *T. cruzi* minicircles (kDNA-*T. cruzi*) [17]. Each sample was tested in a final volume of 20 μl including 5 μl of extracted DNA. The final concentrations of reagents, amplification program, control PCR and electrophoresis were performed as described [46]. A positive result for PCR-XD was the presence of a 330 bp specific band for DNA *T. cruzi*.

### Real-time PCR-xenodiagnosis (qPCR-XD)

To obtain a standard curve to perform the quantification of *T. cruzi*, we used a stock of epimastigote forms of Tulahuén (Tc VI) and Dm28c (Tc I) strains, starting with a known concentration of parasite DNA and performing serial dilutions (1:10) in DNA extracted of FS of negative XD applied under Informed Consent of volunteer without ChD, defining seven points ranging from 10^5^ to 10^-1^ parasite equivalent/ml (par. eq./ml). All calculations were made assuming that *T. cruzi* genetic material has a mass of 200 fg [19]. The standard curve of *T. cruzi* was accepted considering the average of the tests with an efficiency (Eff) of 99.0 %, linearity (Y) of -3.346 and correlation (*R*^*2*^) of 0.999 and on the other hand of human chromosome 12 (X12) with an Eff of 102.4 %, Y of -3.226 and *R*^*2*^ of 0.997. The standard curve of CIE corresponding to X12 was designed to discard false-negative cases caused by the absence of DNA in the evaluated sample due to extraction problems, and to assess whether there was inhibition in the PCR reaction. The primers N1X12 forward (5′-AGC TGG CTA GAC TGT CAT-3′) and N2X12 reverse (5′ CTT TGC CGT TGA AGC TTG-3′) and the probe N3X12 (5'-TGG GAC TTC AGA GTA GGC AGA TCG-3') were used as described [2, 4]. The standard curve for X12 was prepared with a pool of human genomic DNA of 5 individuals with ELISA and IFI-IgG negative for *T. cruzi*. After extraction and purification, total DNA quantification was carried out using AccuBlue™ High Sensitivity dsDNA Quantitation Kit (Biotium Inc., California, USA) in a qPCR instrument Mx3000P (Stratagene, Agilent Technologies Inc., California, USA) as detector devices. The average concentration of total genomic DNA was 3.4 ng/μl. Later, six points of dilution 1:5 in elution buffer Qiagen kit were performed. The concentrations ranged between 3.40 and 0.00108 ng/μl. The standard curve of X12 was maintained at -20 °C until use. The reaction mixture was composed of 2 μl of the sample to be investigated in duplicate, 10 μl of Brilliant Multiplex QPCR Master Mix, 0.5 μl of a 1:500 dilution of a reference dye (ROX), 2 μl of primers N1X12 and N2X12, 0.8 μl of probe N3X12, 0.2 μl of BSA (100×) (BioLabs Inc., New England, UK) and 2.5 μl of molecular biology grade water, in a final reaction volume of 20 μl.

qPCR was applied in the pools of FS obtained by XD to determine the presence and amount of *T. cruzi* amplified by *Tr. infestans* using TaqMan™ detection system in a thermocycler Stratagene Mx3000P (Stratagene, Agilent Technologies, Inc., California, USA) as described [2]. The following controls in duplicate were included in each reaction: water, buffer, pool of FS-DNA extracted of 5 uninfected triatomines as negative control of *T. cruzi* and as positive control a pool of FS-DNA of 5 XD of CChD individuals with parasitemia previously confirmed by positive PCR and XD.

The controls of X12 in duplicate were water, buffer, pool of DNA extracted (previously added to human DNA) of 5 individuals as a positive control and pool DNA of sample XD of 5 individuals as a negative control.

The thermal profile of all qPCR assays consisted in 10 min of pre-incubation at 95 °C and 40 cycles of amplification with one estimate time of reaction of 90 min. The emitted fluorescence was registered at 60 °C at the end of each cycle using the MxPro QPCR Software version 4.10 (Stratagene, Agilent Technologies Inc., California, USA) for Mx3000P System that established the threshold fluorescence value for each assay according to the settings provided in Analysis Term Settings. The fluorescence under threshold was considered negative (No Ct).

### Statistical analyses

The data were analyzed using the Statistical Package for the Social Sciences (SPSS version19.0 Chicago, Illinois, USA) software. The description of the data was performed by tables, percentages, and arithmetic mean. Fisher’s exact test and Chi-square were applied for comparison of qualitative variables. In the quantitative variables, Levene's test was applied to evaluate heteroscedasticity and Kruskal-Wallis test for comparison between the averages of parasite burden. In all statistical tests it was considered statistically significant *P* < 0.05.

## Results and discussion

**Fig. 1:**
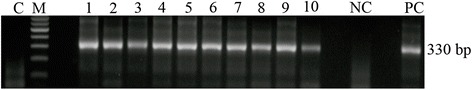
Amplified products of *Trypanosoma cruzi* by PCR in fecal samples of human xenodiagnosis. GelRed™ stained 2 % agarose gel of amplified products of *Trypanosoma cruzi* minicircle kinetoplastid PCR from fecal samples (FS) of triatomines (*Triatoma infestans*) fed on untreated chronic chagasic patients (PCR-XD). Lane C: control; Lane M: 100 bp ladder; Lanes 1-10: PCR-XD positive in ten patients (band of 330 bp specific for *T. cruzi*); Lane NC: FS-DNA extracted from five triatomines free infection; Lane PC: DNA *T. cruzi* Tulahuén strain

XD is a procedure in which the insect vector is used as a biological culture medium for the detection of *T. cruzi* infection [21]. Of the 100 cases evaluated in this study by XD, in 21 it was possible to observe microscopically mobile forms of *T. cruzi* in the FS of triatomines. For the contrary, in 79 cases, the XD was negative. To improve the limited sensitivity of the direct observation, especially in FS with low parasite numbers, molecular diagnostic techniques such as PCR have been directly applied in DNA extracted from FS [5, 18, 20, 40, 47]. In humans and diverse mammal reservoirs, the utility of PCR to detect *T. cruzi* in FS of triatomines fed by XD has been reported [9, 10, 44, 46, 51]. For this reason, the biological samples of this study (DNA extracted of FS used in XD), represent a great advantage in relation to *T. cruzi* DNA extracted from peripheral blood, because even with very low parasitemia, the XD is capable of amplification with an inoculum of at least a living parasite [46], constituting irrefutable evidence of viable *T. cruzi.* In the present study, 100 % of the cases with positive XD and 73.4 % with negative XD, were PCR-XD (Fisher’s exact test: *P* = 0.005) positive (Fig. 1).

Nevertheless, PCR is a qualitative technique that is not useful to quantify *T. cruzi* in peripheral blood or FS of triatomines naturally infected or fed by XD on diverse hosts. In this work, *T. cruzi* levels, by qPCR-XD in FS of triatomines fed by XD in untreated individuals with CChD were quantified by qPCR-XD with the aim of assessing the ability of infection and multiplication of *T. cruzi* in *Tr. infestans* incubated during 90 days post-feeding. Additionally, qPCR in peripheral blood (qPCR-B) was performed for all patients in the study as described [2].

The positivity of qPCR-XD in the XD negative group was 83.5 % (Fisher’s exact test: *P* = 0.037). In Table 1, it is possible to observe that *T. cruzi* quantification by qPCR in the FS of positive cases by XD allowed us to establish that in 42.9 % (9/21 cases) the parasite burden fluctuated between 100 and 1,000 par. eq./ml. In this group, the smallest and largest parasite burden was between 1–10 par. eq./ml (9.5 %) and 1,000–10,000 par. eq./ml (23.8 %). In cases with negative XD, 40.5 % of them (32/79 cases), a parasite burden between 1–10 par. eq./ml was detected. The lowest and highest parasite burden fluctuated between 0.1–1 par. eq./ml (34.2 %) and 100–1,000 par. eq./ml (2.5 %). The average of parasite burden through qPCR-XD in the negative XD group were lower than those of positive XD group: 15 and 752 par. eq./ml, respectively (Kruskal-Wallis test: *χ*^*2*^ = 43.248, *df* = 1, *P* = 0.0001).

**Table 1:** Ranges of parasite burden of *Trypanosoma cruzi* determined by real-time PCR in samples of *Triatoma infestans* used in xenodiagnosis (qPCR-XD) and peripheral blood (qPCR-B) in 100 individuals with chronic Chagas disease classified by positive or negative XD

	Positive XD^a^		Negative XD^b^	
	*n* = 21		*n* = 79	
Ranges parasite burden (parasite equivalent/ml)	qPCR-XD†^c^	qPCR-B	qPCR-XD†^c^	qPCR-B
	*n* (%)	*n* (%)	*n* (%)	*n* (%)
No Ct	0 (0)	0 (0)	13 (16.5)	17 (21.5)
0.1–1	0 (0)	8 (38.1)	27 (34.2)	29 (36.7)
1–10	2 (9.5)	7 (33.3)	32 (40.5)	30 (38.0)
10–100	5 (23.8)	4 (19.0)	5 (6.3)	2 (2.5)
100–1,000	9 (42.9)	1 (4.8)	2 (2.5)	1 (1.3)
1,000–10,000	5 (23.8)	1 (4.8)	0 (0)	0 (0)

†Heteroscedasticity detected: Levene's test, *F* = 187.663, *P =* 0.0001

^a^Chi-square test (qPCR-XD *vs* qPCR-B): *χ* ^*2*^ = 91.82, *df =* 5, *P =* 0.0001

^b^Chi-square test (qPCR-XD *vs* qPCR-B): *χ* ^*2*^ = 6.71, *df* = 5, *P* = 0.1520

^c^Kruskal-Wallis test: *χ* ^*2*^ = 42.48, *df* = 2, *P* = 0.0001

*Abbreviation*: No Ct, No threshold cycle (DNA-*T.cruzi* was not detected)

In relation to qPCR-B, in 21 cases with positive XD, 38.1 % (8 cases) and 33.3 % (7 cases) had parasite burden between 0.1–1 and 1–10 par. eq./ml, respectively. Only 2 cases (9.6 %) had parasite burden greater than 100 par. eq./ml. On the other hand, in the cases with negative XD, 30 cases (38 %) had a parasite burden between 1–10 par. eq./ml, 29 cases (36.7 %) had a parasite burden between 0.1–1 par. eq./ml, a single case had parasite burden between 100–1,000 par. eq./ml. In 17 cases (21.5 %) *T. cruzi* was not detected (Table 1). In the positive XD group, the concordance between the positivity of qPCR-XD and qPCR-B was 100 %; however the parasitemia ranges were different (Chi-square test: *χ*^*2*^ = 91.82, *df* = 5, *P* = 0.0001). In the negative XD group, qPCR-XD detected four cases more than qPCR-B, but the statistical analysis demonstrated that the ranges of parasitemia of qPCR-XD and qPCR-B were similar (Chi-square test: *χ*^*2*^ = 6.71, *df* =5, *P* = 0.1520) (Additional File 1: Table S1).

All samples evidenced amplification of X12 (Ct average: 31.8) so problems in the extraction process (excess or loss of genetic material), unspecific reactions and/or inhibition of qPCR-XD were discarded. The positive control showed amplification of X12.

Are demonstrated that XD is a useful tool to detect living *T. cruzi* in persons with CChD [46], therefore the positivity of PCR-XD and qPCR-XD suggests the presence of viable tripomastigotes naturally amplified by the biological vector. Nevertheless, diverse factors must be considered in the sensitivity of XD: number of boxes used, patient age, capacity of ingestion by the bugs [3, 12, 24, 49], strong diuresis of triatomines with loss of initial inoculum after a large blood meal [14, 32], complexity of the fecal metabolome of triatomines that suggests that it may affect triatomine competence for specific *T. cruzi* strains [1], bacterial microbiota in the triatomine gut [27]; influence of starvation in the development of *T. cruzi* in *Tr. infestans* [31], mortality during the incubation period and differential regulation of parasite populations that shows that some triatomine defense reactions discriminate not only between molecular signals specific for trypanosome infections but also between different strains of *T. cruzi* [25]. Other aspects related with the last point also can be important in the sensitivity of XD: *T. cruzi* genotypes amplified from single and mixed infections [25, 32], transfer of a *T. cruzi* DTU from mixture present in human blood to triatomine vector depends on the specie used in the XD [41, 47], the development of one clonal genotype from mixed infection can be inhibited [43] and that the most important strains belonging to one of the two main DTUs, developed different population densities in the insect vector [48].

## Conclusions

This study allowed the detection and quantification of *T. cruzi* by qPCR-XD in FS of *Tr. infestans* fed on patients with CChD. Nevertheless the different variables related with the sensitivity of XD, the highest parasite burden were observed in positive XD cases. qPCR-XD could be used in different studies related with the complex *T. cruzi*-vector-host interactions.

## Abbreviations

BSA, albumin serum bovine; ChD, chagas disease; DNA, deoxyribonucleic acid; dNTP, deoxynucleotide triphosphate; ELISA, enzyme linked immunosorbent assay; FS, fecal samples; FS-DNA, DNA of fecal samples; IFI, indirect immunofluorescence; kDNA, kinetoplastidic DNA; PBS, phosphate solution buffer; PCR, polymerase chain reaction; PCR-XD, conventional PCR performed in FS of XD; qPCR, real time PCR or quantitative PCR; qPCR-XD, quantitative PCR performed in fecal samples of XD; X12, human chromosome 12; XD, xenodiagnosis.

## Additional file

Additional file 1: Table S1. Xenodiagnosis (XD), PCR-xenodiagnosis (PCR-XD), real-time PCR-xenodiagnosis (qPCR-XD) and real-time PCR-blood (qPCR-B) results in 100 individuals with CChD. The ranged parasite burden are represented by numbers 0–5: **0** (No Ct); **1** (0.1–1 par. eq./ml); **2** (1–10 par. eq./ml); **3** (10–100 par. eq./ml); **4** (100–1,000 par. eq./ml) and **5** (1,000–10,000 par. eq./ml). (DOCX 22 kb)

## References

[1] Antunes LC, Han J, Pan J, Moreira CJ, Azambuja P, Borchers CH (2013). Metabolic signatures of triatomine vectors of *Trypanosoma cruzi* unveiled by metabolomics. PLoS One.

[2] Apt W, Arribada A, Zulantay I, Rodríguez J, Saavedra M, Muñoz A (2013). Treatment of Chagas' disease with itraconazole: electrocardiographic and parasitological conditions after 20 years of follow-up. J Antimicrob Chemother.

[3] Barreto AC, Marsden PD, Cuba CC, Alvarenga NJ (1978). Preliminary study on the use of *Dipetalogaster maximus* (Uhler, 1894) (Triatominae) in the xenodiagnostic technic in the chronic form of Chagas' disease. Rev Inst Med Trop Sao Paulo.

[4] Bravo N, Muñoz C, Nazal N, Saavedra M, Martínez G, Araya E (2012). Real-Time PCR in faecal samples of *Triatoma infestans* obtained by xenodiagnosis: proposal for an exogenous internal control. Parasit Vectors..

[5] Breniere SF, Bosseno MF, Tellería J, Carrasco R, Vargas F, Yaksic N (1995). Field application of polymerase chain reaction diagnosis and strain typing of *Trypanosoma cruzi* in Bolivian triatomines. Am J Trop Med Hyg.

[6] Britto C, Cardoso MA, Marques P, Fernandes O, Morel CM (1999). Polymerase chain reaction detection: new insights into the diagnosis of chronic Chagas disease. Mem Inst Oswaldo Cruz.

[7] Britto CC (2009). Usefulness of PCR-based assays to assess drug efficacy in Chagas disease chemotherapy: value and limitations. Mem Inst Oswaldo Cruz.

[8] Brumpt E (1914). Le xenodiagnostic. Application au diagnostic de quelques infections parasitaries et en particulier a la trypanosomose de Chagas. Bull Soc Pat Exot.

[9] Campos R, Acuña-Retamar M, Botto-Mahan C, Ortiz S, Cattán P, Solari A (2007). Susceptibility of *Mepraia spinolai* and *Triatoma infestans* to different *Trypanosoma cruzi* strains from naturally infected rodent hosts. Acta Trop.

[10] Campos R, Botto-Mahan C, Ortiz S, Coronado X, Solari A (2010). Temporal fluctuation of infection with different *Trypanosoma cruzi* genotypes in the wild rodent *Octodon degus*. Am J Trop Med Hyg.

[11] Castro C, Candido M, Silveira S (2004). Estudo comparativo entre o xenodiagnóstico artificial realizado inmediatamente e quatro horas após a coleta de sangue. Rev Bras Med Trop.

[12] Castro CN, Alves MT, Macêdo VO (1983). Importância da repetição do xenodiagnóstico para avaliação da parasitemia na fase crônica da doença de Chagas. Rev Soc Bras Med Trop.

[13] Cecere MC, Cardinal MV, Arrabal JP, Moreno C, Gürtler RE (2015). *Microcavia australis* (Caviidae, Rodentia), a new highly competent host of *Trypanosoma cruzi* I in rural communities of northwestern Argentina. Acta Trop..

[14] Cirano R, Zeledón R (1964). Observaciones sobre capacidad alimenticia y respiración de *Triatoma infestans* y *Rodhnius prolixus* (Hemiptera: Reduvidae). Rev Biol Trop..

[15] Correa JP, Bacigalupo A, Fontúrbel FE, Oda E, Cattan PE, Solari A (2015). Spatial distribution of an infectious disease in a small mammal community. Naturwissenschaften.

[16] Coura JR, Borges-Pereira J (2011). Chronic phase of Chagas disease: why should it be treated? A comprehensive review. Mem Inst Oswaldo Cruz.

[17] Degrave W, Fragoso SP, Britto C, van Heuverswyn H, Kidane GZ, Cardoso MA (1988). Peculiar sequence organization of kinetoplast DNA minicircles from *Trypanosoma cruzi*. Mol Biochem Parasitol.

[18] Dorn PL, Flores J, Brahney B, Gutiérrez A, Rosales R, Rodas A (2001). Comparison of polymerase chain reaction on fresh tissue samples and fecal drops on filter paper for detection of *Trypanosoma cruzi* in *Rhodnius prolixus*. Mem Inst Oswaldo Cruz.

[19] Duffy T, Bisio M, Altcheh J, Burgos JM, Diez M, Schijman A (2009). Accurate Real-Time PCR strategy for monitoring bloodstream parasitic loads in Chagas disease patients. PLoS Negl Trop Dis.

[20] Egaña C, Vergara F, Campos R, Ortiz S, Botto C, Solari A (2014). *Trypanosoma cruzi infection* in *Mepraia gajardoi* and *Mepraia spinolai*: the effect of feeding nymphs from the field. Am J Trop Med Hyg.

[21] Enriquez GF, Bua J, Orozco MM, Wirth S, Schijman AG, Gürtler RE (2014). High levels of *Trypanosoma cruzi* DNA determined by qPCR and infectiousness to *Triatoma infestans* support dogs and cats are major sources of parasites for domestic transmission. Infect Genet Evol..

[22] Del P Fernández M, Cecere MC, Lanati LA, Lauricella MA, Schijman AG, Gürtler RE (2014). Geographic variation of *Trypanosoma cruzi* discrete typing units from Triatoma infestans at different spatial scales. Acta Trop..

[23] Franco Y, Rassi A, Rodrigues A, García H, Rassi GG, García da Silva I, Rassi A, Rodrigues A, García H, Rassi GG (2002). Correlation among the positivity of the artificial xenodiagnosis and the amount of blood and triatomines used in the exam, in chronic chagasic patients. Rev Soc Bras Med Trop.

[24] Freitas JL (1961). Diagnóstico de laboratório da moléstia de Chagas. Bol. Of. Sanit. Panamer..

[25] García ES, Genta FA, de Azambuja P, Schaub GA (2010). Interactions between intestinal compounds of triatomines and* Trypanosoma cruzi*. Trends Parasitol.

[26] González Cappa SM, Chiale P, del Prado GE, Katzin AM, de Martini GW, de Isola ED (1980). Isolation of a strain of *Trypanosoma cruzi* from a patient with chronic Chagas cardiomyopathy and its biological characterization. Medicina (B Aires).

[27] Gumiel M, da Mota FF, Rizzo V de S, Sarquis O, De Castro DP, Lima MM (2015). Characterization of the microbiota in the guts of *Triatoma brasiliensis* and *Triatoma pseudomaculata* infected by *Trypanosoma cruzi* in natural conditions using culture independent methods.. Parasit Vectors.

[28] Gürtler RE, Cardinal MV (2015). Reservoir host competence and the role of domestic and commensal hosts in the transmission of *Trypanosoma cruzi*. Acta Trop..

[29] Herrera CP, Licon MH, Nation CS, Jameson SB, Wesson DM (2015). Genotype diversity of *Trypanosoma cruzi* in small rodents and *Triatoma sanguisuga* from a rural area in New Orleans, Louisiana. Parasit Vectors..

[30] INE, Instituto Nacional de Estadísticas, Chile. Compendio Estadístico 2010. 1.2 Estadísticas Demográficas. 50 pp*.* http://www.ine.cl/canales/menu/publicaciones/compendio_estadistico/pdf/2010/1.2estdemograficas.pdf. Accessed 28 June 2016.

[31] Kollien AH, Schaub GA (1998). The development of *Trypanosoma cruzi* (Trypanosomatidae) in the reduviid bug *Triatoma infestans* (Insecta): influence of starvation. J Eukaryot Microbiol.

[32] Kollien AH, Schaub GA (2000). The development of *Trypanosoma cruzi* in triatominae. Parasitol Today.

[33] Lima VS, Jansen AM, Messenger LA, Miles MA, Llewellyn MS (2014). Wild *Trypanosoma cruzi* I genetic diversity in Brazil suggests admixture and disturbance in parasite populations from the Atlantic Forest region. Parasit Vectors..

[34] Marcet PL, Duffy T, Cardinal MV, Burgos JM, Lauricella MA, Levin MJ (2006). PCR-based screening and lineage identification of *Trypanosoma cruzi* directly from faecal samples of triatomine bugs from northwestern Argentina. Parasitology.

[35] Mejía-Jaramillo AM, Agudelo-Uribe LA, Dib JC, Ortiz S, Solari A, Triana-Chávez O (2014). Genotyping of *Trypanosoma cruzi* in a hyper-endemic area of Colombia reveals an overlap among domestic and sylvatic cycles of Chagas disease. Parasit Vectors..

[36] MINSAL. Ministerio de Salud, Chile. Norma general técnica. Control y prevención de la enfermedad de Chagas. 2014. 97 pp. http://web.minsal.cl/sites/default/files/NORMA%20TECNICA_CHAGAS_FINAL.pdf. Accessed 28 June 2016

[37] MINSAL. Ministerio de Salud, Chile. Segunda Encuesta Nacional de Salud. ENS Chile. 2009-2010. 1064 pp. http://web.minsal.cl/portal/url/item/bcb03d7bc28b64dfe040010165012d23.pdf. Accessed 28 June 2016.

[38] Morillo CA, Marin-Neto JA, Avezum A, Sosa-Estani S, Rassi A, Rosas F (2015). Randomized trial of benznidazole for chronic Chagas' cardiomyopathy. N Engl J Med.

[39] Muñoz C, Zulantay I, Apt W, Ortiz S, Schijman AG, Bisio M (2013). Evaluation of nifurtimox treatment of chronic Chagas disease by means of several parasitological methods. Antimicrob Agents Chemother.

[40] Oda E, Solari A, Botto-Mahan C (2014). Effects of mammal host diversity and density on the infection level of *Trypanosoma cruzi* in sylvatic kissing bugs. Med Vet Entomol.

[41] Ortiz S, Zulantay I, Apt W, Saavedra M, Solari A (2015). Transferability of *Trypanosoma cruzi* from mixed human host infection to *Triatoma infestans* and from insects to axenic culture. Parasitol Int.

[42] Peterson JK, Bartsch SM, Lee BY, Dobson AP (2015). Broad patterns in domestic vector-borne *Trypanosoma cruzi* transmission dynamics: synanthropic animals and vector control. Parasit Vectors..

[43] Pinto AS, de Lana M, Bastrenta B, Barnabé C, Quesney V, Noël S (1998). Compared vectorial transmissibility of pure and mixed clonal genotypes of *Trypanosoma cruzi* in *Triatoma infestans*. Parasitol Res.

[44] Pizarro JC, Lucero D, Stevens L (2007). A method for the identification of guinea pig blood meal in the Chagas disease vector *Triatoma infestans*. Kinetoplastid Biol Dis..

[45] Prata A (2001). Clinical and epidemiological aspects of Chagas disease. Review. Lancet Infect Dis..

[46] Saavedra M, Zulantay I, Apt W, Martínez G, Rojas A, Rodríguez J (2013). Chronic Chagas disease: PCR-xenodiagnosis without previous microscopic observation is a useful tool to detect viable *Trypanosoma cruzi*. Biol Res.

[47] Santana RA, Magalhães LK, Magalhães LK, Prestes SR, Maciel MG, da Silva GA (2014). *Trypanosoma cruzi* strain TcI is associated with chronic Chagas disease in the Brazilian Amazon. Parasit Vectors..

[48] Schaub GA (1989). *Trypanosoma cruzi*: quantitative studies of development of two strains in small intestine and rectum of the vector *Triatoma infestans*. Exp Parasitol.

[49] Schenone H (1999). Xenodiagnosis. Mem Inst Oswaldo Cruz.

[50] WHO. World Health Organization. Chagas disease: certification of interruption of transmission in Chile. Wkly Epidemiol Rec 2000; http://www.who.int/docstore/wer/pdf/2000/wer7502.pdf. Accessed 28 June 2016.

[51] Zulantay I, Apt W, Valencia C, Torres A, Saavedra M, Rodríguez J (2011). Detection of *Trypanosoma cruzi* in untreated chronic chagasic patients is improved by using three parasitological methods simultaneously. J Antim Chemother.

