# Supplementary material for: Quantification by real-time PCR of Trypanosoma cruzi DNA in samples of Triatoma infestans used in xenodiagnosis of chronic Chagas disease patients
Source: Parasit Vectors. 2016 Jul 4;9:382. doi: 10.1186/s13071-016-1664-5 (PMC4932745; doi:10.1186/s13071-016-1664-5)
Supplement: Additional file 1: Table S1. — Xenodiagnosis (XD), PCR-xenodiagnosis (PCR-XD), real-time PCR-xenodiagnosis (qPCR-XD) and real-time PCR-blood (qPCR-B) results in 100 individuals with CChD. The ranged parasite burden are represented by numbers 0–5: 0 (No Ct); 1 (0.1–1 par. eq./ml); 2 (1–10 par. eq./ml); 3 (10–100 par. eq./ml); 4 (100–1,000 par. eq./ml) and 5 (1,000–10,000 par. eq./ml). (DOCX 22 kb) [file 13071_2016_1664_MOESM1_ESM.docx]

**Additional file 1: Table S1**. Xenodiagnosis (XD), PCR-xenodiagnosis (PCR-XD), real-time PCR-xenodiagnosis (qPCR-XD) and real-time PCR-blood (qPCR-B) results in 100 individuals with CChD. The ranged parasite burden are represented by numbers 0–5: **0** (No Ct); **1** (0.1–1 par. eq./ml); **2** (1–10 par. eq./ml); **3** (10–100 par. eq./ml); **4** (100–1,000 par. eq./ml) and **5** (1,000–10,000 par. eq./ml).

| Number case | XD | PCR-XD | qPCR-XD | qPCR-B |
| --- | --- | --- | --- | --- |
| 1 | (+) | (+) | 4 | 2 |
| 2 | (+) | (+) | 3 | 3 |
| 3 | (+) | (+) | 4 | 2 |
| 4 | (+) | (+) | 5 | 4 |
| 5 | (+) | (+) | 5 | 3 |
| 6 | (+) | (+) | 4 | 3 |
| 7 | (+) | (+) | 5 | 5 |
| 8 | (+) | (+) | 4 | 2 |
| 9 | (+) | (+) | 3 | 2 |
| 10 | (+) | (+) | 4 | 3 |
| 11 | (+) | (+) | 4 | 2 |
| 12 | (+) | (+) | 4 | 2 |
| 13 | (+) | (+) | 3 | 1 |
| 14 | (+) | (+) | 2 | 1 |
| 15 | (+) | (+) | 4 | 1 |
| 16 | (+) | (+) | 3 | 1 |
| 17 | (+) | (+) | 2 | 1 |
| 18 | (+) | (+) | 4 | 2 |
| 19 | (+) | (+) | 5 | 1 |
| 20 | (+) | (+) | 3 | 1 |
| 21 | (+) | (+) | 5 | 1 |
| 22 | (-) | (+) | 0 | 1 |
| 23 | (-) | (+) | 0 | 1 |
| 24 | (-) | (+) | 1 | 0 |
| 25 | (-) | (+) | 1 | 0 |
| 26 | (-) | (+) | 2 | 1 |
| 27 | (-) | (+) | 2 | 2 |
| 28 | (-) | (+) | 0 | 2 |
| 29 | (-) | (+) | 0 | 1 |
| 30 | (-) | (+) | 3 | 1 |
| 31 | (-) | (+) | 1 | 1 |
| 32 | (-) | (+) | 2 | 1 |
| 33 | (-) | (+) | 1 | 1 |
| 34 | (-) | (+) | 0 | 1 |
| 35 | (-) | (+) | 1 | 1 |
| 36 | (-) | (+) | 1 | 1 |
| 37 | (-) | (+) | 1 | 1 |
| 38 | (-) | (+) | 2 | 1 |
| 39 | (-) | (+) | 0 | 0 |
| 40 | (-) | (+) | 1 | 0 |
| 41 | (-) | (+) | 1 | 1 |
| 42 | (-) | (+) | 0 | 0 |
| 43 | (-) | (+) | 0 | 2 |
| 44 | (-) | (+) | 1 | 0 |
| 45 | (-) | (+) | 0 | 1 |
| 46 | (-) | (+) | 1 | 1 |
| 47 | (-) | (+) | 1 | 1 |
| 48 | (-) | (+) | 0 | 1 |
| 49 | (-) | (+) | 3 | 0 |
| 50 | (-) | (+) | 0 | 0 |
| 51 | (-) | (+) | 0 | 0 |
| 52 | (-) | (+) | 1 | 0 |
| 53 | (-) | (+) | 1 | 0 |
| 54 | (-) | (+) | 2 | 0 |
| 55 | (-) | (+) | 3 | 0 |
| 56 | (-) | (+) | 3 | 2 |
| 57 | (-) | (+) | 4 | 3 |
| 58 | (-) | (+) | 1 | 0 |
| 59 | (-) | (+) | 0 | 0 |
| 60 | (-) | (+) | 1 | 2 |
| 61 | (-) | (+) | 1 | 2 |
| 62 | (-) | (+) | 2 | 0 |
| 63 | (-) | (+) | 2 | 3 |
| 64 | (-) | (-) | 2 | 2 |
| 65 | (-) | (+) | 2 | 4 |
| 66 | (-) | (-) | 2 | 2 |
| 67 | (-) | (-) | 2 | 1 |
| 68 | (-) | (-) | 2 | 2 |
| 69 | (-) | (-) | 2 | 1 |
| 70 | (-) | (+) | 2 | 2 |
| 71 | (-) | (+) | 1 | 1 |
| 72 | (-) | (+) | 1 | 1 |
| 73 | (-) | (-) | 2 | 2 |
| 74 | (-) | (-) | 4 | 2 |
| 75 | (-) | (+) | 2 | 2 |
| 76 | (-) | (+) | 1 | 2 |
| 77 | (-) | (+) | 2 | 2 |
| 78 | (-) | (+) | 1 | 2 |
| 79 | (-) | (+) | 2 | 2 |
| 80 | (-) | (-) | 2 | 2 |
| 81 | (-) | (-) | 2 | 2 |
| 82 | (-) | (-) | 1 | 1 |
| 83 | (-) | (-) | 2 | 2 |
| 84 | (-) | (+) | 2 | 2 |
| 85 | (-) | (+) | 2 | 2 |
| 86 | (-) | (-) | 2 | 2 |
| 87 | (-) | (-) | 2 | 2 |
| 88 | (-) | (-) | 2 | 2 |
| 89 | (-) | (-) | 1 | 1 |
| 90 | (-) | (-) | 1 | 2 |
| 91 | (-) | (-) | 2 | 1 |
| 92 | (-) | (+) | 1 | 0 |
| 93 | (-) | (+) | 2 | 2 |
| 94 | (-) | (-) | 2 | 2 |
| 95 | (-) | (-) | 1 | 1 |
| 96 | (-) | (+) | 2 | 1 |
| 97 | (-) | (-) | 2 | 1 |
| 98 | (-) | (+) | 2 | 2 |
| 99 | (-) | (-) | 1 | 1 |
| 100 | (-) | (+) | 3 | 2 |
